# Carrageenan and insulin resistance in humans: a randomised double-blind cross-over trial

**DOI:** 10.1186/s12916-024-03771-8

**Published:** 2024-11-26

**Authors:** Robert Wagner, Janine Buettner, Martin Heni, Louise Fritsche, Stephanie Kullmann, Moritz Wagmüller, Andreas Peter, Hubert Preissl, Jürgen Machann, Reiner Jumpertz von Schwartzenberg, Andreas L. Birkenfeld, Ulrich-Frank Pape, Gerrit van Hall, Peter Plomgaard, Hans-Ulrich Häring, Andreas Fritsche, Kelsey N. Thompson, Reinhild Klein, Norbert Stefan

**Affiliations:** 1 Department of Internal Medicine IV, Division of Endocrinology, Diabetology and Nephrology, University Hospital of Tübingen, Tübingen, Germany; 2 Institute for Diabetes Research and Metabolic Diseases (IDM) of the Helmholtz Centre Munich at the University of Tübingen, Tübingen, Germany; 3 German Center for Diabetes Research (DZD), Neuherberg, Germany; 4 Department of Endocrinology and Diabetology, Medical Faculty and University Hospital, Heinrich Heine University, Moorenstr 5, Düsseldorf, 40225 Germany; 5 Institute for Clinical Diabetology, German Diabetes Center, Leibniz Center for Diabetes Research at Heinrich Heine University, Düsseldorf, Germany; 6 Department of Hepatology and Gastroenterology, Charité Universitätsmedizin, Berlin, Germany; 7 Institute for Diabetes and Obesity, Helmholtz Diabetes Center, Helmholtz Zentrum München, German Research Center for Environmental Health (GmbH), Neuherberg, Germany; 8 Department of Internal Medicine I, Ulm University Hospital, Ulm, Germany; 9 Institute for Clinical Chemistry and Pathobiochemistry, Department for Diagnostic Laboratory Medicine, University Hospital of Tübingen, Tübingen, Germany; 10 Section On Experimental Radiology, Department of Diagnostic and Interventional Radiology, University Hospital of Tübingen, Tübingen, Germany; 11 Department of Internal Medicine and Gastroenterology, Asklepios Klinik St. Georg, Hamburg, Germany; 12 Department of Biomedical Sciences, University of Copenhagen, Copenhagen, Denmark; 13 Department of Clinical Biochemistry, Rigshospitalet, Copenhagen, Denmark; 14 Department of Biostatistics, Microbiome Analysis Core, Harvard T.H. Chan School of Public Health, Boston, USA; 15 Department of Internal Medicine II, Division of Haematology, Oncology, Immunology and Rheumatology, University Hospital of Tübingen, Tübingen, Germany; 16 Institute of Pharmaceutical Sciences, Department of Pharmacy and Biochemistry; Interfaculty Centre for Pharmacogenomics and Pharma Research at the Eberhard Karls University Tübingen, Tübingen, Germany

**Keywords:** Carrageenan, Intestinal permeability, Gut microbiome, Insulin sensitivity, Emulsifiers, Type 2 diabetes

## Abstract

**Background:**

The potential impact of specific food additives, common in Western diets, on the risk of developing type 2 diabetes is not well understood. This study focuses on carrageenan, a widely used food additive known to induce insulin resistance and gut inflammation in animal models, and its effects on human health.

**Methods:**

In a randomised, double-blind, placebo-controlled, cross-over trial conducted at a university hospital metabolic study centre, 20 males (age 27.4 ± 4.3 years, BMI 24.5 ± 2.5 kg/m^2^) participated. The intervention involved oral intake of carrageenan (250 mg) or placebo in the morning and in the evening and each intervention lasted 2 weeks. The primary outcome measured was insulin sensitivity (using oral glucose tolerance test [OGTT] and hyperinsulinaemic-euglycaemic clamp). Additional end-points included whole body and hepatic insulin sensitivity, MRI-measured brain inflammation and insulin resistance, intestinal permeability (via lactulose-mannitol test and plasma zonulin levels), and gut microbiome composition. Immune-cell activation and pro-inflammatory cytokine release from peripheral blood mononuclear cells were measured.

**Results:**

Overall insulin sensitivity did not show significant differences between the treatments. However, interactions between BMI and treatment were observed (OGTT-based insulin sensitivity index: *p*=0.04, fasting insulin resistance: *p*=0.01, hepatic insulin sensitivity index: *p*=0.04). In overweight participants, carrageenan exposure resulted in lower whole body and hepatic insulin sensitivity, a trend towards increased brain inflammation, and elevated C-reactive protein (CRP) and IL-6 levels compared to placebo. Additionally, carrageenan was associated with increased intestinal permeability. *In vitro* natural killer (NK-)cell activation and increased pro-inflammatory cytokine release were found after carrageenan exposure in the participant’s peripheral blood mononuclear cells.

**Conclusions:**

These findings suggest that carrageenan, a common food additive, may contribute to insulin resistance and subclinical inflammation in overweight individuals through pro-inflammatory mechanisms in the gut. Further investigation into the long-term health impacts of carrageenan and other food additives is warranted.

**Trial registration:**

NCT02629705.

**Supplementary Information:**

The online version contains supplementary material available at 10.1186/s12916-024-03771-8.

## Background

The global spread of ‘Western-style’ diet strongly contributes to increasing diabetes prevalence worldwide. This diet consists of ultra-processed foods with high amounts of calories from fat and carbohydrates, abundant saturated fatty acids, and a low intake of fibres, which are known to increase fat mass [1] and, thereby, may predominantly drive the type 2 diabetes epidemic. Furthermore, sugar-sweetened beverages, particularly high fructose, may impair glucose metabolism independently of changes in body fat mass [2]. It is unknown if other dietary components of a Western-style diet also play a role in this process. The food additive carrageenan is widely used by the food industry to enhance food texture [3], particularly in dairy (e.g. ice cream, milk beverages) and meat (e.g. sausages, processed dried meat) products. Its hydrocolloid and gel-forming properties are being used as gelling agents and emulsifiers. Due to its potential to act as a fat substitute, it is often used in low calorie and low-fat food products. Structurally, carrageenans are large polysaccharides consisting of sulphated D-galactose and anhydro-galactose moieties. The anhydro-galactose content and the position of the ester-sulphate bonds modulate their solubility and define various classes of carrageenans. While high molecular-weight carrageenan is considered toxicologically safe, poligeenans, hydrolysis degradation products of carrageenan, cause intestinal inflammation and ulcerations in mice and exhibit carcinogenic properties [4]. The average daily carrageenan intake increased from 45 mg in the 1970s to over 250 mg at the beginning of the twenty-first century in some countries [3]. Epidemiologically, an increase in carrageenan consumption has been associated with the incidence of breast cancer [5]. An association of increased carrageenan consumption with higher incidence of diabetes has been found in a recent population-based European study [6]. In vivo studies in mice have shown that carrageenan added to drinking water induces glucose intolerance [7] and exacerbates unfavourable effects of high fat diet on glycemia [8]. However, clinical studies investigating carrageenan’s effect on glycaemic traits in humans have not been performed. Therefore, this study investigated the effects of carrageenan supplementation on insulin resistance (both peripheric and brain), on subclinical inflammation, gut permeability, and gut microbiome composition.

## Methods

### Trial design and participants

Young non-obese men without known chronic disease were recruited to participate in a placebo-controlled, randomised, double-blind cross-over study to test the effects of carrageenan intake vs placebo, each over a period of 2 weeks, on insulin sensitivity and metabolism. The trial was performed at the Study Centre of the University Hospital Tübingen, Department of Internal Medicine IV (Endocrinology and Diabetology), Tübingen, Baden-Württemberg, Germany, between October 2015 and December 2016. To prevent bias from the menstrual cycle on insulin sensitivity, only males were recruited. Further inclusion criterion was a body-mass index (BMI) range of 18.5 and 29.9 kg/m^2^. Participants with chronic disease, any ongoing medication, regular alcohol consumption over 30 g per day, or shift work were excluded. The flow of the study is shown in Fig. S1. The participants were randomised to the treatments (Fig. S2) using a computerised block-randomisation with a block-size of 10. Randomisation sequences have been generated by an independent person, and the test substances have been allocated according to this sequence by the University Pharmacy. Participants and investigators were blinded to the assignment of interventions. Each participant underwent two consecutive expositions, one with each test substance over 14 days. There was a washout-period of 21–35 days between the two exposures. As a fasted state was required for the OGTT, the hyperinsulinaemic-euglycaemic clamp, and the whole-body magnetic resonance imaging (MRI), these diagnostic procedures were performed on three consecutive days during the last 3 days of each exposition interval (visits 2, 3, and 4 and visits 6, 7, and 8). Participants were instructed to carry on their previous lifestyles and do not change their diets throughout the whole study period. The study was approved by the Ethical Committee of the Medical Faculty of the University of Tübingen, Germany. All participants provided written informed consent. The study was performed in accordance with the Declaration of Helsinki and was registered at clinicaltrials.org (NCT02629705). There were no changes in the trial methods after its commencement.

### Intervention

The intervention substance (test substance) consisted of 250 mg carrageenan or placebo (mannitol/aerosil) filled into standard capsules. To fill up the full volume of the capsules, both verum and placebo preparations were topped up with mannitol/aerosol (Fagron GmbH, Glinde, Germany and Caesar & Loretz GmbH, Hilden, Germany). Carrageenan (E 407) was purchased in a 25 kg container from a food industry supplier (A. Schmidt & Co. GmbH, Hamburg, Germany). Gelling property was given as 403 g/cm^2^ for water and 700 g/cm^2^ for milk. Other attributes were not specified on the product sheet. Participants were asked to add the test substance to unsweetened yogurt twice daily, once in the morning and once in the evening.

### Outcome measures and their measurements

The primary outcome measure was insulin sensitivity assessed by oral glucose tolerance test (OGTT). The co-primary outcome was insulin sensitivity assessed by hyperinsulinaemic-euglycaemic clamp (M-value). The secondary outcomes were: endogenous glucose production measured by tracer-method, cerebral insulin sensitivity, intrahepatic triglyceride content, and glycaemia during OGTT. Other outcome measures were intestinal permeability, intestinal microbiome constitution, and markers of systemic inflammation.

#### Diagnostic procedures (study days)

Identical diagnostic assessments were performed at the end of both exposition phases. Each of these comprised three study days (visits 2, 3, and 4 and visits 6, 7, and 8). The first assessment days (visit 2 and 6) lasted from 8:00 am to 11:00 am and comprised a brief physical examination, fasting blood sample collection, and an OGTT. Intake of the test substance was carried on at the end of these assessments (at 11:00, and in the evening). The second assessment days (visits 3 and 7) comprised hyperinsulinaemic-euglycaemic clamps starting at 7:00 am and lasting to approximately 1:00 pm. Test substance intake was resumed afterward. The third assessment days (visits 4 and 8) comprised whole-brain MRI and whole-body MRI examinations starting at 7:00 am. The assessments concluded with the lactulose-mannitol test, which lasted over 4 h until approximately 1:00 pm. Stool samples could be provided on the second or third assessment days.

#### Oral glucose tolerance test

After an overnight fast, participants underwent standardized 75 g OGTT on the first day of the diagnostic procedures after test substance expositions (visits 2 and 6). Blood samples were obtained via antecubital indwelling venous catheters at fasting and at minutes 30, 60, 90, and 120.

#### Hyperinsulinaemic-euglycaemic glucose tracer clamp

Participants underwent hyperinsulinaemic-euglycaemic clamps with the administration of 6·6-_2_H^2^ labelled glucose. Blood was obtained from an indwelling venous catheter. The upper extremity was heated by an electronic heating cuff to provide arterialized blood samples. After a bolus, isotonic saline with 0.4% glucose solution of > 98% deuterated glucose (Profil Institute, Neuss, Germany) was administered continuously according to a body-weight adapted scheme [9]. The hyperinsulinaemic clamp started at minute 120 with an insulin bolus over 10 min followed by a body surface area standardized insulin infusion rate of 25 mU∙m^−2^∙min^−1^. Plasma glucose levels were measured with a glucometer (EKF, Germany) during the clamp and held constant at 5 mmol/L by the administration of a 2% deuterated 20% glucose solution. Endogenous glucose production (EGP) was measured from samples obtained before insulin stimulation at minutes 100, 110, and 120 and during insulin stimulation at 250, 260, and 270 as previously described [10]. One participant missed the clamp during his second phase. Clamp-based whole-body insulin sensitivity was calculated as the ratio of the mean glucose infusion rate and mean plasma insulin over the last 30 min of the clamp. Hepatic insulin sensitivity was determined as percentage suppression of mean EGP at minutes 250, 260, and 270 during hyperinsulinemia compared to baseline, divided by the mean insulin level during the same period of the clamp.

#### Whole-brain MRI

Whole-brain MRI was obtained by using a 3 Tesla scanner (Siemens PRISMA) with a 20-channel head coil. Three participants did not undergo this measurement since they had dental retainers or large tattoos. Cerebral free water content (FW) was estimated as a proxy for brain inflammation. This method is based on MR-visible-proton density (PD) with an acquisition time of 14 min [11]. As obesity is particularly related to hypothalamic inflammation [11], FW values of the hypothalamus were further evaluated in relationship to FW values of the total grey matter. To quantify cerebral insulin sensitivity, cerebral blood flow was measured by arterial spin labelling before and 30 min after the administration of nasal insulin [12].

#### Body fat mass and distribution and liver fat content

Total body, subcutaneous, and visceral fat mass were measured by MRI and liver fat content by ^1^H-MR spectroscopy, as previously described. The measurements were performed in both assessment blocks, after expositions with the test substances.

#### Lactulose mannitol intestinal permeability test

After the OGTT, the participants underwent lactulose-mannitol tests to measure intestinal permeability. The test was standardized according to the recommendation of Sequeira et al. [13]. In brief, participants ingested 250 mL water solution of 5 g mannitol and 15 mL lactulose over 5 min. They drank 250 mL water over the next hour. Urine collection was performed between minutes 150 and 240 in 19 participants.

#### Faecal sample treatment and DNA sequencing

Faecal samples were collected on between day 2 and 3 of the assessment studies, frozen after collection, and kept frozen at – 80 °C until metagenome analysis. Whole genome sequencing was performed at the Alkek Center for Metagenomics and Microbiome Research, Baylor College of Medicine, Houston, Texas, USA. After DNA extraction, samples were processed using the HiSeqX – 3 Gb pipeline.

#### Bioinformatic processing and downstream statistical analysis

Raw sequencing reads were processed by the Harvard T.H. Chan School of Public Health Microbiome Analysis Core using the bioBakery whole metagenome shotgun workflow curated by the Huttenhower lab (http://huttenhower.sph.harvard.edu/biobakeryworkflows). Briefly, raw samples were first run through KneadData v0.6.0 for quality control and contaminant removal. Final read count per sample averaged 24,405,955 (min: 16,979,144; max: 31,783,415); no samples were lost to post-sequencing QC. Next, MetaPhlAn 2 v2.6.0 [14] was used to assign taxonomy to each sample, using species-specific marker genes. Finally, functional profiling was performed using HUMAnN 2 v0.11.0. We applied omnibus, blockwise, and feature-wise statistical models to characterise the potential effect of carrageen on structure and function of the gut microbiome. Univariate PERMANOVAs on Bray–Curtis dissimilarities tested for compositional alteration after intervention.

#### Cellular and humoral immunological reactions

Peripheral blood mononuclear cells (PBMC) were isolated from heparinised blood that had been obtained during fasting, at recruitment, and after the two consecutive expositions according to standard procedures [15] and then incubated with or without antigens for 2 days. Carrageenan was added to the cultures at concentrations ranging from 1 mg/ml to 0.1 μg/ml. Tetanus toxoid and BCG were used as recall-antigen known to stimulate T helper type 2 cells and T helper type 1 cells or macrophages, respectively. Proliferation of PBMC was determined on day 7 by thymidine-uptake [15]. Cytokines (interleukin [IL]−1, interleukin-5, interleukin-6, interleukin-10, interleukin-13, interferon [IFN]-g, tumour necrosis factors [TNF] a and b) were measured in the PBMC supernatants after culturing them with the antigens for 7 days using antibody pairs and recombinant cytokines as standards (Pharmingen, San Diego CA, USA).

For the determination of activated immune cells by flow cytometry, the PBMC were incubated with or without the antigens for 24 h. The activation marker CD69 was determined on ™, B, and natural killer (NK) cells using FastImmune™CD4/CD69/CD3, FastImmune™CD8/CD69/CD3, FastImmune™CD19/CD69/CD45, and FastImmune™CD56/CD69/CD45 (Becton–Dickinson, San Jose, CA).

Patients’ sera were tested for antibodies to Carrageenan by an in-house ELISA [16]. Carrageenan was used at a concentration of 40 μg/ml; patients’ sera were diluted 1:200 for the demonstration of antibodies to the IgG and IgM type and 1:200 for the detection of IgA antibodies. Secondary peroxidase-conjugated anti-human IgG, IgM, and IgA antibodies (Dianova, Hamburg, Germany) were applied at a dilution of 1:2.000.

### Study outcomes

The primary study outcome was insulin sensitivity as estimated by the Matsuda index, and the co-primary outcome was insulin sensitivity estimated from hyperinsulinaemic clamp. Secondary outcomes comprised hepatic insulin sensitivity, brain insulin sensitivity, brain inflammation, body fat mass and distribution, liver fat content, and glycemia. Additional end points were intestinal permeability, systemic inflammation markers, and intestinal microbiome composition.

### Laboratory measurements

Plasma insulin was determined by an immunoassay with the ADVIA Centaur XP Immunoassay System (Siemens Healthineers, Eschborn, Germany). Triglycerides (TGs) and total, HDL, and low-density lipoprotein (LDL) cholesterol levels as well as alanine aminotransferase (ALT), aspartate aminotransferase (AST), and gamma-glutamyl transpeptidase (GGT) activities were measured using the ADVIA XPT clinical chemical analyser (Siemens Healthineers, Eschborn, Germany). Plasma concentrations of total non-esterified fatty acids (NEFA) were measured with an enzymatic method (WAKO Chemicals, Neuss, Germany) on the latter instrument. HbA1c was measured with Tosoh glycohaemoglobin analyser HLC-723G8 (Tosoh Bioscience Tokyo Japan). Plasma zonulin was measured from fasting samples obtained on the day of OGTT by an ELISA (Immunddiagnostik, Bensheim, Germany). The glucose tracer enrichment was measured using liquid chromatography tandem mass spectrometry.

For the carbohydrate absorption test, 500 μL of urine was desalted with Amberlite MB-3 resin in the acetate form, and protein was removed with sulfosalicylic acid. Using meso-erythritol and turanose as internal standards, the sugars were separated, analysed, and quantified by HPLC with pulsed electrochemical detection (Dionex, Idstein, Germany): chromatography module: 250 × 40 mm Carbopac PA-1 column (Dionex); eluent 150 mmol NaOH; flow; 1 ml/min. Results were expressed as the percentage recovery of the ingested dose of the sugars. Intestinal permeability was expressed as the ratio of lactulose and mannitol recovery.

#### Gut microbiome analysis

Raw sequencing reads were processed by the Harvard T.H. Chan School of Public Health Microbiome Analysis Core using the bioBakery whole metagenome shotgun workflow curated by the Huttenhower lab (http://huttenhower.sph.harvard.edu/biobakeryworkflows).

#### Statistical analyses

Statistical evaluation was performed according to Wellek and Blettner’s recommendations for cross-over studies [17]. In brief, all outcomes were first tested for potential carry-over effects by comparing the intra-individual sums of the outcome variables between the placebo–carrageenan and the carrageenan–placebo groups by a standard non-paired *t-test*. The main study effect was tested by comparing within-subject differences between the two exposition sequences (placebo–carrageenan vs. carrageenan–placebo, two-sided non-paired *t-test*, *⍺* = 0.05). The number of participants required was determined based on the effect size in the animal model in the work by Bhattacharyya et al. [7]. The study was designed to detect an effect of carrageenan in humans that is up to 5 times smaller than in animals (*d* = 5.8/5 = 1.16). With this effect size, a total of 42 examinations was estimated, yielding 21 participants in the cross-over design. Post hoc analyses were computed using mixed linear regression models testing BMI × treatment interactions and further adjustment for BMI, treatment, the treatment sequence as fixed effects, and the participant as random effect. For mixed linear regression, the lme4 library was used. All computations were performed in R V3.4.

## Results

### Insulin sensitivity, circulating inflammatory markers, and intestinal permeability

**Fig. 1:**
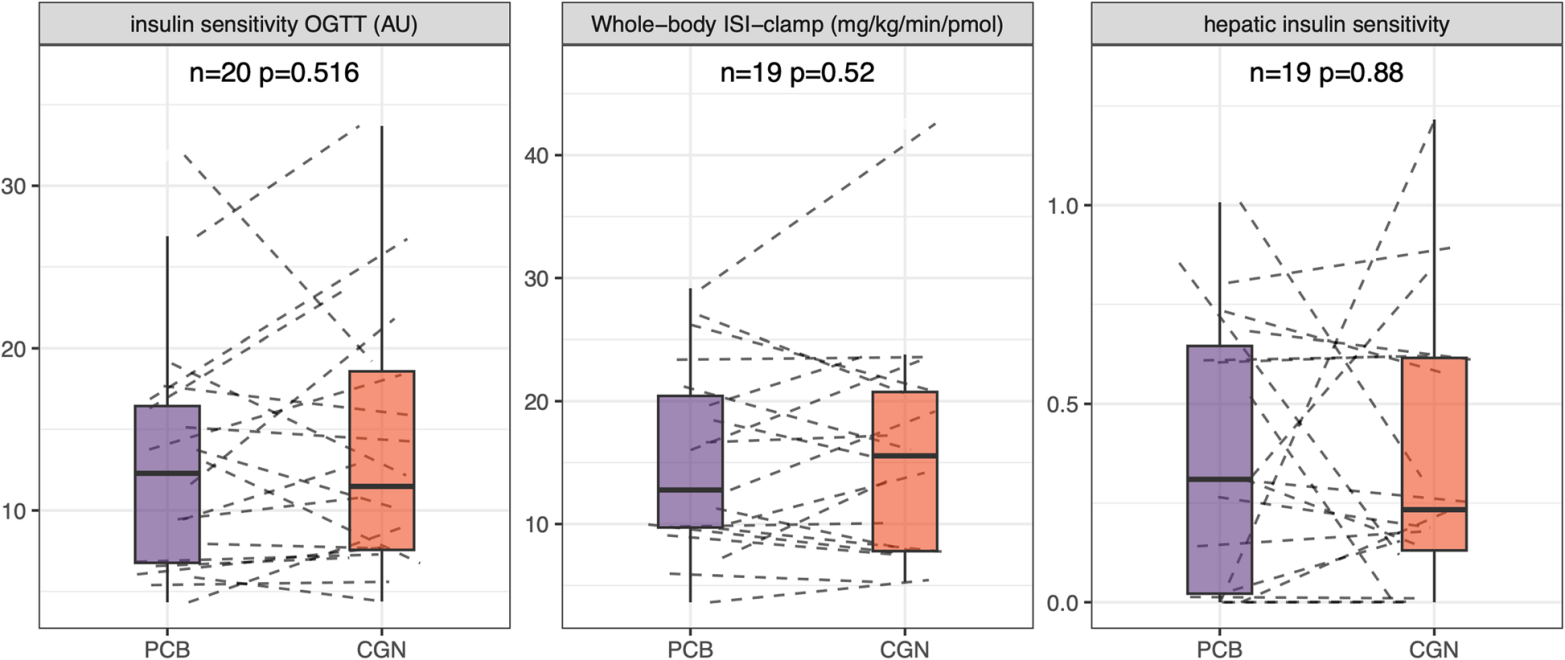
**A**–**C** Differences in whole-body insulin sensitivity (**A**) and organ-related insulin sensitivity (predominantly skeletal muscle (**B**), liver (**C**)) after placebo (PCB) and carrageenan (CGN) administration in the study. Box plots indicate median values with thick horizontal lines. The upper and lower hinges correspond to the first and third quartiles (the 25th and 75th percentiles). The whiskers extend from the hinges to the lowest/highest value that is within 1.5 * interquartile range (IQR) of the hinge. Dashed lines show individual changes between treatment phases. The *p*-values were determined comparing within-subject differences between the two exposition sequences (placebo–carrageenan vs. carrageenan–placebo) with two-sided non-paired *t*-tests, and *n* indicates the numbers (differences) tested

Characteristics of the study participants at randomisation are shown in Table S1. None of the participants fulfilled the criteria of metabolic syndrome according to National Cholesterol Education Program Adult Treatment Panel III [18]. Carrageenan supplementation was well tolerated. There were no exposure-related adverse events. Results for all pre-specified study endpoints are shown in Table S2. The primary outcome was insulin sensitivity assessed from an OGTT, representing whole-body insulin sensitivity. The co-primary end-point was whole-body insulin sensitivity measured during hyperinsulinaemic-euglycaemic clamp, predominantly representing skeletal muscle insulin sensitivity in the young and non-obese subjects. None of these variables showed differences between treatments (*n* = 20 pairs for the OGTT and *n* = 19 pairs for clamp, *p* = 0.52 for both, Fig. 1A, B). Hepatic insulin sensitivity was not different between exposures (*n* = 19 pairs, *p* = 0.88, see Fig. 1C).

**Fig. 2:**
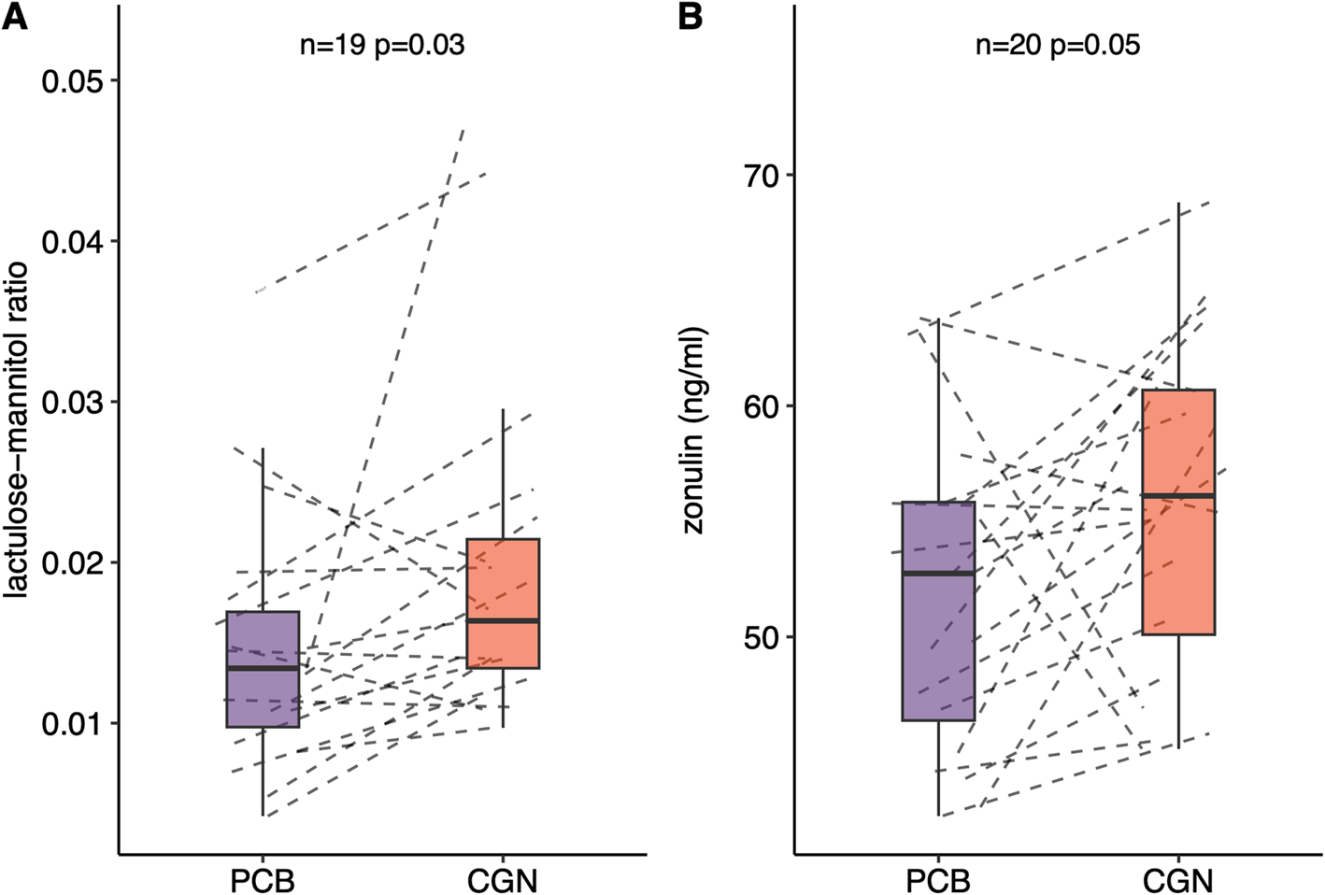
**A**, **B** Intestinal permeability expressed by lactulose-mannitol ratio (**A**) and plasma zonulin levels (**B**) after treatments with placebo (PCB) and carrageenan (CGN). Box plots indicate median values with thick horizontal lines. The upper and lower hinges correspond to the first and third quartiles (the 25th and 75th percentiles). The whiskers extend from the hinges to the lowest/highest value that is within 1.5 * interquartile range (IQR) of the hinge. Dashed lines show individual changes between treatment phases. The *p*-values were determined comparing within-subject differences between the two exposition sequences (placebo–carrageenan vs. carrageenan–placebo) with two-sided non-paired *t*-tests, and *n* indicates the numbers (differences) tested

Hypothalamic insulin response, representing brain insulin sensitivity (*N* = 17, *p* = 0.09), hypothalamic inflammation (*N* = 17, *p* = 0.2), and hepatic triglyceride content (*p* = 0.6), also did not differ between treatments. The lactulose-mannitol ratio was elevated during carrageenan exposure, showing an increased intestinal permeability during carrageenan intake (*N* = 19, *p* = 0.03, Fig. 2A).

There was no difference in C-reactive protein (CRP) and interleukin-6 (IL-6) levels between the treatments (both *p* > 0.5). No carry-over effects were detected (*p* > 0.05).

#### Post hoc* analyses*

To follow-up on the findings of increased intestinal permeability after high carrageenan exposition, plasma zonulin levels after consumption of the treatments were measured in all participants who completed the study. Circulating zonulin was higher after carrageenan compared to placebo exposure (55.9 vs 52.1 ng/l, *p* = 0.05, Fig. 2B).

**Fig. 3:**
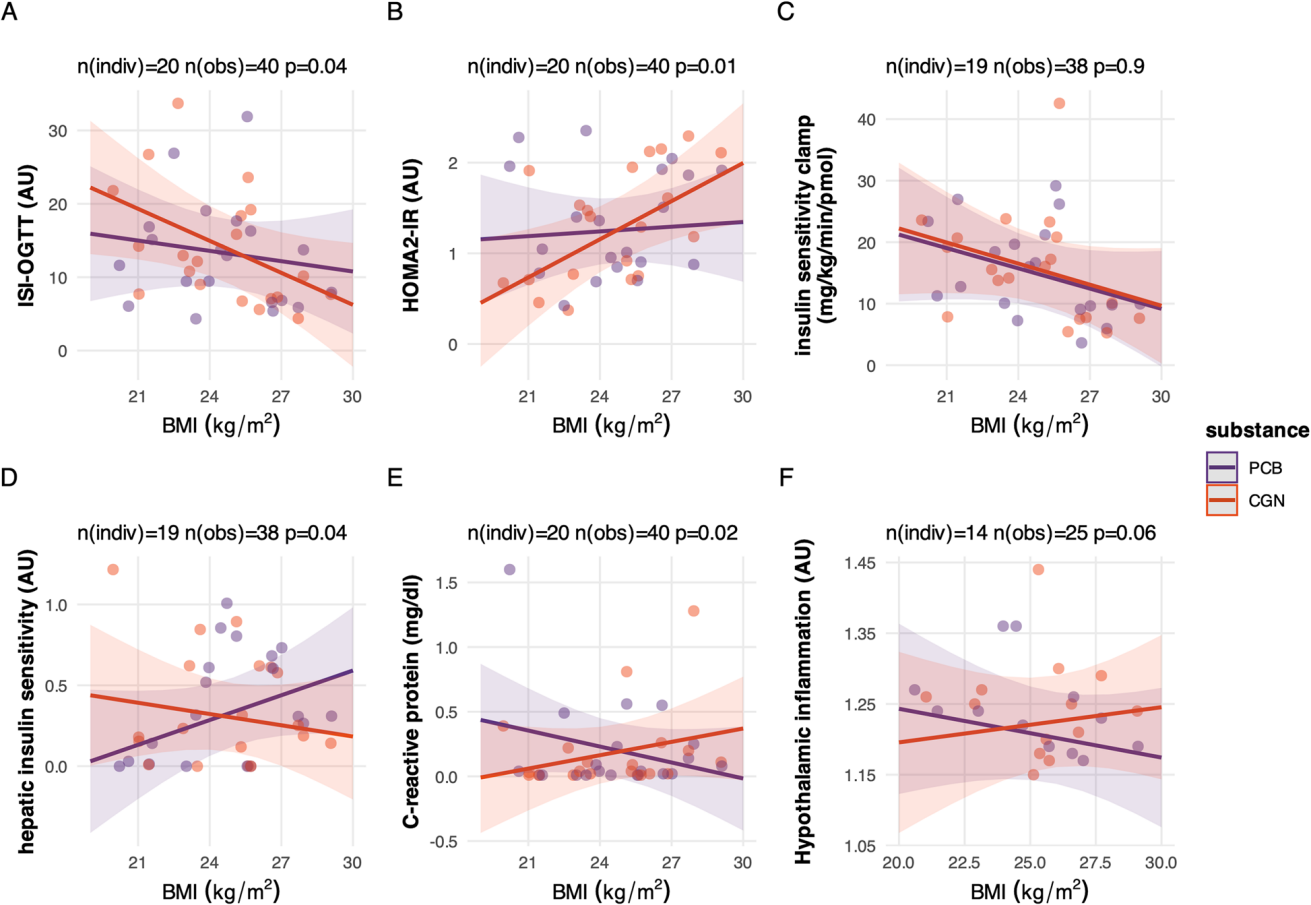
**A**–**F** Interaction between BMI and treatment (placebo–PCB, carrageenan–CGN) on predefined study endpoints. Interaction plots show the modelled association between BMI and whole-body insulin sensitivity (OGTT (Matsuda index (**A**)), HOMA2-IR (**B**), insulin sensitivity of glucose disposal (clamp, M/I) (**C**), hepatic insulin sensitivity (**D**), C-reactive protein (**E**), and hypothalamic inflammation (**F**) for the placebo and carrageenan treatments in the study. The *p*-values are provided for BMI × treatment interaction terms in mixed models. AU, arbitrary units

Studies in mice, which were mostly published after initiation of the present study, indicated that intestinal barrier dysfunction is predominantly induced by a high fat diet, hyperglycaemia, and in obesity [19–21]. As only young non-obese males (mean BMI 24.5, range 20.1 to 29.1 kg/m^2^) were included in the study, systemic effects of carrageenan on glucose metabolism and subclinical inflammation may not be easily detectable. Therefore, the modulation of the study outcomes by BMI was investigated in an ancillary analysis by testing BMI × treatment interactions on pre-specified endpoints (Fig. 3).

These interaction analyses showed lower whole-body insulin sensitivity during the OGTT (*p* = 0.04, Fig. 3A) and higher insulin resistance estimated from fasting glucose and insulin levels (HOMA2-IR; *p* = 0.01, Fig. 3B) for carrageenan with higher BMI. Additional interactions suggested lower hepatic insulin sensitivity with higher BMI levels (*N* = 19, *p* = 0.04, Fig. 3D) and a similar trend for higher hypothalamic inflammation (*N* = 17, *p* = 0.06, Fig. 3F), while no effect on skeletal muscle insulin sensitivity was observed (*N* = 19, *p* = 0.90, Fig. 3C), with carrageenan treatment in overweight subjects. In addition, there was a BMI x treatment interaction on CRP (*p* = 0.02, Fig. 3E) and IL-6 (*p* = 0.02) levels.

#### Effects of carrageenan on cellular and humoral immune reactions

**Fig. 4:**
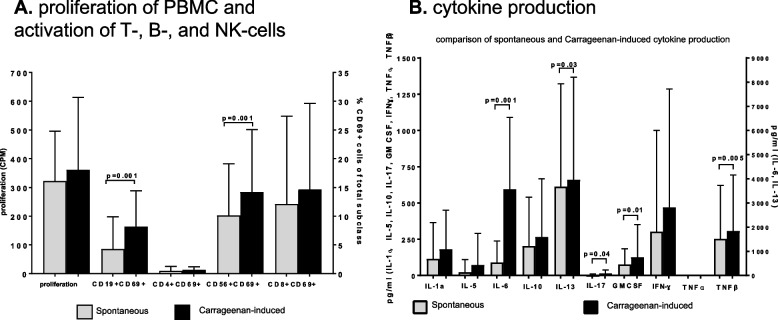
**A**, **B** In vitro analysis of peripheral blood mononuclear cells (PBMCs) from 15 healthy individuals before carrageenan exposure. **A** The proliferation of PBMCs and activation (CD69-expression) of T helper cells (CD4 +), cytotoxic T cells (CD8 +), B cells (CD19 +), and NK cells (CD56 +) via carrageenan. **B** The carrageenan-induced cytokine production

In vitro incubation assays of carrageenan with PBMCs of participants before carrageenan treatment triggered CD19 + B and CD56 + NK cell activation. Furthermore, induction of the cytokines IL-6, IL-13, IL-17, TNF-beta, and GMCSF was observed (Fig. 4).

The pairwise comparison of PBMCs in participants after placebo vs. after carrageenan treatments in vivo showed a trend for carrageenan-induced activation of CD19 + B cells which did not abate in the second phase of the cross-over study (*p* = 0.06), while the carrageenan-induced activation of CD56 + NK cells was suggestive of carryover effects (*p* = 0.06).

Cytokine production assays showed increased tetanus-toxoid-induced IL-13 production after carrageenan treatments (*p* = 0.01, *p* = 0.1); however, other IL-13 induction tests showed carry-over effects (carrageenan-induced *p* = 0.003, spontaneous *p* = 0.004, BCG-induced *p* = 0.04). TNF-ß production was somewhat lower in the carrageenan group (*p* = 0.04 with BCG-stimulation), albeit a trend for carry-over effects was seen (*p* = 0.09). There was no difference between placebo and carrageenan in IL-6 and IL-17 production (data not shown).

The investigation of immunoglobulin A, M, and G antibodies against carrageenan did not show significant differences between treatment phases (*p* > 0.27).

#### Gut microbiome composition

For each individual, their top representative taxa in both exposition phases (blocks A and B) of the study are visualised (Fig. S3). Gut ecosystems were dominated by the usual taxa of Bacteroidetes, Firmicutes, and Prevotella. Little change between the sampling windows was observed. Only small increases in Bray–Curtis dissimilarity were observed over the course of the study, as highlighted by a principal coordinate analysis, with the exception of *Prevotella copri* carries, samples from the same individual ordinate closely together. No significant associations between the beta diversity (Bray–Curtis) and the clinical covariates were observed using a PERMANOVA analysis (FDR *q*-val < 0.2).

## Discussion

According to the United States Food and Drug Administration, carrageenan belongs to substances that are ‘generally regarded as safe’. However, effects of augmented carrageenan intake on human metabolism had not been rigorously evaluated in clinical studies. Results from animal studies about safety of carrageenan are inconclusive [4]. In rodents, carrageenan has been implicated in ulcerative colitis [22], liver cirrhosis [23], insulin resistance, and dysglycemia [7, 8]. While long-term carrageenan exposition did not reduce survival in non-human primates [24], high doses induced intestinal inflammation [25]. Supporting these animal studies, evidence from the current work indicates that carrageenan increases intestinal permeability in humans. Using the clinical gold-standard lactulose-mannitol test, increased exposure to carrageenan resulted in higher lactulose absorption. Interestingly, this effect of carrageenan inducing gut inflammation was not associated with any clinically relevant symptoms, indicating that these negative effects of increased carrageenan exposure may go unnoticed for a substantial time.

To follow-up on a possible effect of carrageenan inducing intestinal permeability, a protein secreted by intestinal epithelial cells in response to dietary or microbiota stimuli was examined. Zonulin is known to reduce the expression of intestinal tight junction proteins, to induce T cell-mediated mucosal inflammation and to control the transmigration of immune cells from the gut into other parts of the body [26]. Tight junctions between epithelial cells of the small intestine can be lost upon epithelial injury. This allows bacteria and toxins to enter the blood stream, potentially leading to sepsis and organ failure. The breakdown of the intestinal barrier enables a proinflammatory environment including differentiation of autoreactive Th17 cells and other T helper cells [27] Furthermore, macromolecules with antigen characteristics may elicit a local or systemic immune response. The pathological symptoms and reactions that are consequences of increased intestinal permeability is a condition called leaky gut syndrome [28]. Zonulin is overexpressed in the intestinal mucosa of subjects with celiac disease [29]. Zonulin levels are elevated in patients with type 1 diabetes and their relatives [30]. Furthermore, in patients with type 1 diabetes, elevated circulating zonulin correlates with increased intestinal permeability and altered gene expression of intestinal tight junction proteins. In addition, zonulin upregulation was detected during the pre-diabetic stage and preceded the onset of type 1 diabetes [30]. In the current study, carrageenan intake also led to increased zonulin levels. However, it should be noted that commercially available ELISA kits, including the one used in this study, may identify various proteins related to zonulin, not exclusively the permeability-regulating protein [31]. Unlike its hydrolysis product, poligeenan, high molecular-weight carrageenan is not absorbed from the intestine in relevant amounts [4]. However, uptake into Peyer patches and mesenteric lymph nodes via macrophages can occur, according to studies in rats with gavage of radiolabelled carrageenan [32]. Physiologic intraluminal degradation of carrageenan to poligeenan depends on the acidity of gastric environment and could, therefore, interact with diet and medication [33]. Furthermore, several bacterial species exhibit carrageenase activity, suggesting a possible interaction of carrageenan degradation with individual microbiome composition. Simulation of the gastric environment showed that around 10% of carrageenan is degraded to hydrolysation products with molecular weights < 100 kDa [34]. The proinflammatory effects of degraded carrageenans are well documented. In the present study, discrete changes in B cell activation and IL-13 production were found during increased carrageenan intake. A robust activation of B and NK cells was detected with direct in vitro exposition of PBMC to carrageenan, and also an increased production of proinflammatory cytokines (IL-6, IFNɣ, TNF⍺) was observed. Interestingly, induction of a mixed Th2/Th17 cytokine profile has been shown to be associated with the development of more severe disease pathogenesis in allergic disorders [35]. Those proinflammatory effects of carrageenan may, therefore, lead to an impaired intestinal epithelial cell function via Toll-like receptor 4 signalling [36, 37] and/or direct effects on circulating immune cells [38, 39].

Oral carrageenan exposure induced insulin resistance in mice and carrageenan inhibited insulin-induced increases in phospho-(Ser473)-Akt and PI3K activity in vivo in mouse liver and in human HepG2 cells. Despite these findings, a 2-week period of elevated carrageenan intake in the current study did not affect whole-body or tissue-specific insulin sensitivity. This finding was puzzling, considering the observed effects with increased carrageenan intake on intestinal permeability and inflammation. The regulation of insulin sensitivity is complex and connected to an extensive metabolic cross-talk between several organs including the liver, adipose tissue, pancreas, skeletal muscle, and brain. Therefore, a relatively short-term increased intake of carrageenan may not be sufficient to modulate such a complex phenotype. Furthermore, with a mean (SD) age of 27.4 (± 4.3) years and BMI of 24.5 (± 2.5) kg/m^2^, the participants in this study were mostly metabolically healthy. Studies in mice suggest that intestinal barrier dysfunction is predominantly induced by high-fat diet, hyperglycaemia, and obesity [19–21]. This led to the hypothesis that increased carrageenan intake could differently impact insulin sensitivity in participants with lower and higher BMI. Indeed, participants with higher BMI experienced a lower whole-body insulin sensitivity and lower hepatic insulin sensitivity during increased carrageenan intake. A trend toward elevated hypothalamic inflammation was also observed. Importantly, increased carrageenan intake did not interact with BMI on insulin sensitivity of glucose disposal. As intestinal inflammation is considered to predominantly regulate insulin sensitivity of liver and brain, and to a much lesser extent of skeletal muscle [40], the absent effect of increased carrageenan intake on skeletal muscle insulin sensitivity was expected. In the interaction with higher BMI, increased carrageenan intake was also associated with elevated circulating CRP and IL-6 levels.

Emulsifiers, detergent-like molecules that are ubiquitous components of processed foods, were found to be linked with diabetes in a human cohort [6] and, with metabolic syndrome, increased pro-inflammatory potential and gut microbiota encroachment and altered species composition in mice [41]. Therefore, carrageenan-related alterations of gut microbiome composition were also tested in the current study. Gut ecosystems were dominated by the taxa Bacteroidetes, Firmicutes, and Prevotella. Little change between the sampling windows was observed. Only small increases in Bray–Curtis dissimilarity were observed over the course of the study, with the exception of Prevotella copri carriers. Samples from the same individual ordinate closely together, which is in line with previous literature [42].

Limitations of the study include that only young, healthy males were recruited. Also, the relatively short exposition periods could have precluded the detection of longer-term effects on metabolism such as hepatic triglyceride content.

The data indicate that carrageenan acts in synergism with obesity and possibly other factors, such as an altered microbiome, in at-risk individuals to further disrupt the intestinal barrier, exaggerate systemic inflammation, and increase insulin resistance.

## Conclusions

This study provides evidence that increased carrageenan intake can disrupt intestinal barrier function in humans. Although short-term carrageenan treatment did not impact whole-body insulin sensitivity in young, metabolically healthy participants, interactions with BMI suggest effects of carrageenan treatment on insulin sensitivity in those who have a higher BMI. The results warrant caution with carrageenan-containing foods, especially in individuals who are prone to develop type 2 diabetes. Further studies are necessary to test carrageenan’s effects in a population at increased risk.

## Supplementary Information

Additional file 1. Figures S1-S3, Tables S1-S2. FigS1- Participant flow. FigS2 – Study design. FigS3 – Faecal microbiome analysis. TabS1 – Participant characteristics. TabS2 – Study endpoints after treatments.

## Data Availability

No datasets were generated or analysed during the current study.

## Abbreviations

ALT: Alanine aminotransferase
AST: Aspartate aminotransferase
BMI: Body mass index
CGN: Carrageenan
CRP: C-reactive protein
EGP: Endogenous glucose production
GGT: Gamma-glutamyl transpeptidase
HOMA: Homeostasis model assessment
IQR: Interquartile range
OGTT: Oral glucose tolerance test
NEFA: Non-esterified fatty acids
NK cells: Natural killer cells
PBMC: Peripheral blood mononuclear cells
PCB: Placebo
