# Supplementary material for: Carrageenan and insulin resistance in humans: a randomised double-blind cross-over trial
Source: BMC Med. 2024 Nov 26;22:558. doi: 10.1186/s12916-024-03771-8 (PMC11590543; doi:10.1186/s12916-024-03771-8)
Supplement: Supplementary file 1 — Additional file 1. Figures S1-S3, Tables S1-S2. FigS1- Participant flow. FigS2 – Study design. FigS3 – Faecal microbiome analysis. TabS1 – Participant characteristics. TabS2 – Study endpoints after treatments. [file 12916_2024_3771_MOESM1_ESM.docx]

Additional file 1 for:

**Carrageenan and insulin resistance in humans: a randomized double-blind cross-over trial**

Robert Wagner *et al.*

*Corresponding author. Email: [robert.wagner@uni-duesseldorf.de](mailto:robert.wagner@uni-duesseldorf.de)

**This PDF file includes:**

Figures S1 to S3

Tables S1 to S2

Figure S1

Participant flow in the study

Figure S2

Scheme of the randomized controlled crossover design. V0 denotes screening visit, V1 start of study medication, V2, 3, 4 assessment visits (Block A) after the first phase, V5 start of study medication in the second phase and V6, 7, 8 assessment visits analogous to V2, 3, 4 (Block B) after the second phase.

Figure S3

Interindividual diversity in stool microbiome samples obscures any potential impact of carrageen use on the composition of the gut microbiome. A) For each individual their top representative taxa in both exposition phases (block A and B) of the study are visualized. Gut ecosystems were dominated by the usual taxa of Bacteroidetes, Firmicutes, and Prevotella. Little change between the sampling windows was observed, as expected. B) Only small increases in Bray-Curtis dissimilarity was observed over the course of the study in our subjects, as highlighted by a principal coordinate analysis, with the exception of *Prevotella copri* carriers, samples from the same individual ordinate closely together, which is in line with previous literature (Lloyd-Price et al, Nature 2019) (C) No significant associations between the beta diversity (Bray-Curtis) and our clinical covariates were observed using a PERMANOVA analysis (FDR q-val < 0.2).


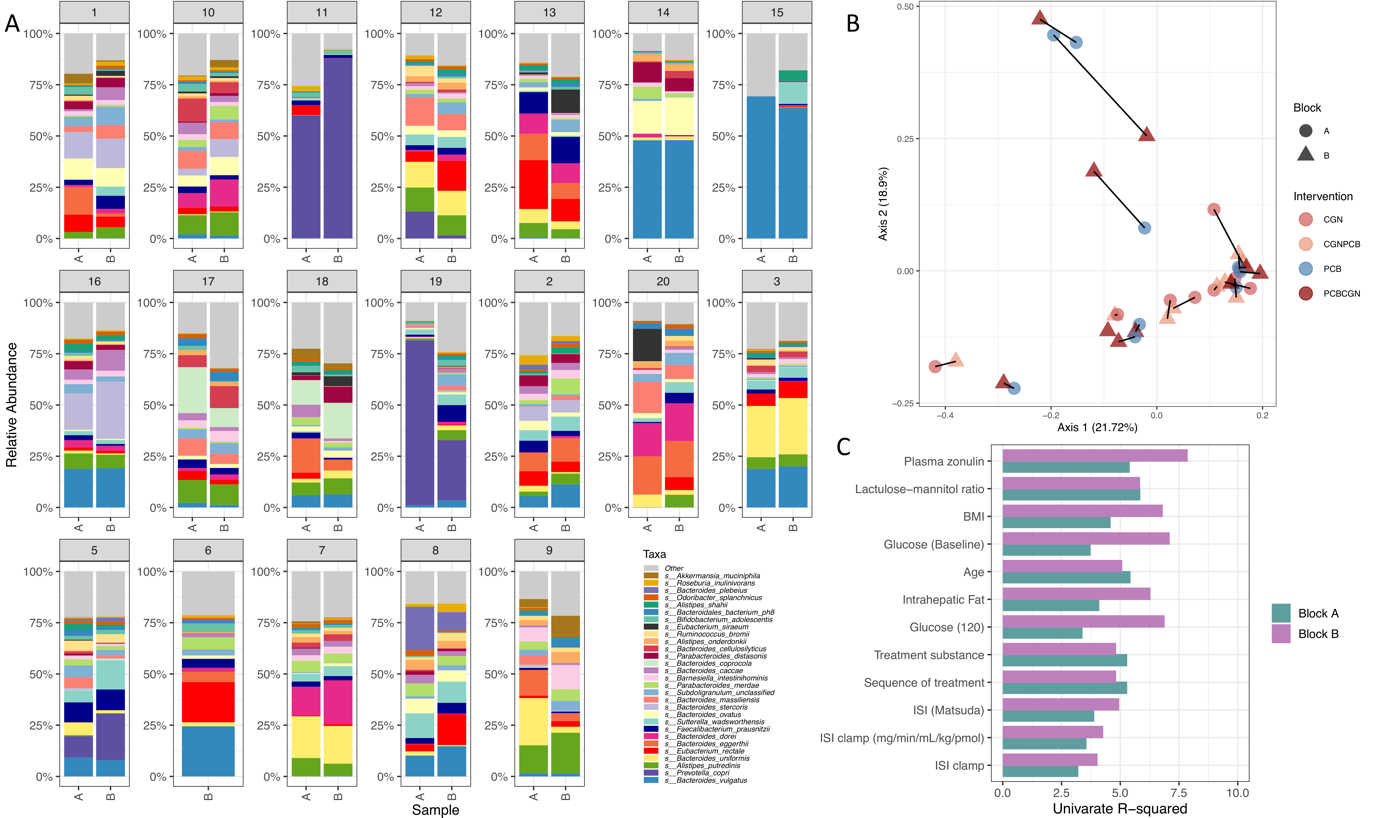


Table S1

Characteristics of the study participants at randomization

|  | Carrageenan first (N=20) | Placebo first  (N=20) | p-value |
| --- | --- | --- | --- |
| Age (years) | 29.1 (4.33) | 27.4 (3.27) | 0.34 |
| Height (cm) | 180.8 (4.04) | 182.4 (5.15) | 0.45 |
| Weight (kg) | 84.17 (10.78) | 80.76 (9.51) | 0.46 |
| BMI (kg/m^2^) | 25.72 (2.88) | 24.37 (2.14) | 0.25 |
| Waist circumference (cm) | 89.4 (6.67) | 86.21 (5.19) | 0.25 |
| Hip circumference (cm) | 96.87 (8.21) | 96.2 (8.46) | 0.86 |
| Waist-to-hip ratio | 0.92 (0.03) | 0.9 (0.08) | 0.44 |
| Body surface area (m^2^) | 2.03 (0.13) | 2.01 (0.13) | 0.72 |

Table S2

Study endpoints after placebo and carrageenan expositions

|  | n_1_ | Placebo | n_2_ | Carrageenan | p-value* |
| --- | --- | --- | --- | --- | --- |
| ISI_OGTT_ (arbitrary units) | 20 | 12.9 ± 2 | 20 | 13.7 ± 2 | 0.5 |
| Whole-body ISI_clamp_ (mg/kg/min/pmol) | 19 | 15.1 ± 2 | 19 | 15.9 ± 2 | 0.5 |
| EGP basal (µmol min^-1^ kg^-1^) | 19 | 11.8 ± 0.3 | 19 | 11.7 ± 0.4 | 0.7 |
| EGP clamp (µmol min^-1^ kg^-1^) | 19 | 1.83 ± 0.4 | 19 | 1.81 ± 0.4 | 0.9 |
| Hepatic insulin sensitivity (AU) | 19 | 0.379 ± 0.08 | 19 | 0.367 ± 0.08 | 0.9 |
| Hepatic ISI basal Vangipurapu (AU) | 19 | 4734 ± 232 | 19 | 4643 ± 193 | 0.7 |
| Brain insulin resistance | 14 | 1.11 ± 0.05 | 14 | 1.03 ± 0.04 | 0.09 |
| Hypothalamic inflammation | 11 | 1.24 ± 0.02 | 11 | 1.26 ± 0.02 | 0.2 |
| Hepatic fat content (%) | 20 | 3.42 ± 1 | 20 | 3.28 ± 0.8 | 0.6 |
| Glycemia (AUC glucose, mmol 120min l^-1^) | 20 | 13 ± 0.7 | 20 | 13.3 ± 0.5 | 0.6 |
| Total body fat mass_MRT_ (liter) | 18 | 21.9 ± 2 | 18 | 22.2 ± 2 | 0.5 |
| Visceral fat mass_MRT_ (liter) | 18 | 2.58 ± 0.3 | 18 | 2.73 ± 0.3 | 0.3 |
| intestinal permeability  (lactulose-mannitol ratio) | 19 | 0.0147 ± 0.002 | 19 | 0.0196 ± 0.002 | 0.03 |
| zonulin (ng ml^-1^) | 20 | 52.1 ± 2 | 20 | 55.9 ± 2 | 0.05 |

Mean ± standard errors. ISI: insulin sensitivity index. EGP: endogenous glucose production. Unpaired t-test comparing the within-subject differences between the two exposition sequences (Placebo-Carrageenan and Carrageenan-Placebo), also see methods.
